# Circulating Apoptotic Progenitor Cells in Patients with Congestive Heart Failure

**DOI:** 10.1371/journal.pone.0003238

**Published:** 2008-09-18

**Authors:** Dael Geft, Shmuel Schwartzenberg, Ori Rogowsky, Ariel Finkelstein, Jacob Ablin, Sofia Maysel-Auslender, Dov Wexler, Gad Keren, Jacob George

**Affiliations:** Department of Cardiology, Tel Aviv Sourasky Medical Center, Tel Aviv, Israel; University of Giessen Lung Center, Germany

## Abstract

**Background:**

Circulating CD34+ endothelial progenitor cells (EPCs) are capable of differentiating into mature endothelial cells to assist in angiogenesis and vasculogenesis. We sought to quantify the numbers of apoptotic progenitors in patients with congestive heart failure.

**Methods and Results:**

Peripheral blood mononuclear cells were isolated by Ficoll density-gradient from 58 patients with various degrees of heart failure and 23 matched controls. Apoptosis in progenitor CD34+ cells was assessed using the Annexin V-PE/PI detection kit, and FACS analysis was performed with triple staining for CD34, annexin-V and propidium iodide. The percentage of early and late apoptotic progenitor cells was determined in the subject groups and was correlated with clinical characteristics. While there was no significant difference in total CD34 positive cells or early apoptotic progenitors between control subjects and CHF patients (p = 0.42) or between severe and mild/moderate CHF groups (p = 0.544), there was an elevated number of late apoptotic progenitors in the severe CHF group compared with the mild/moderate CHF group (p =  0.03). Late apoptotic progenitors were significantly increased in CHF patients as compared to matched controls. There was also an inverse correlation between late apoptotic progenitors and ejection fraction (r = −0.252, p = 0.028) as well as a positive association with NYHA class (r = 0.223, p = 0.046).

**Conclusion:**

Severe heart failure patients exhibited higher numbers of late apoptotic progenitors, and this was positively associated with NYHA class and negatively correlated with ejection fraction. This finding may shed light on the numerous factors governing the pathophysiology of CHF.

## Introduction

Over the past few decades, researchers as well as clinicians have made great strides in understanding the pathophysiological mechanisms of heart failure. Whereas heart failure was once thought of as a series of symptoms simply due to a poorly functioning heart, it is now understood to be a syndrome whose causes are both multifactorial and complex [1]. Several diverse mechanisms contribute to this syndrome including structural and functional abnormalities of the heart, vascular disease, biological and neurohormonal factors, oxidative stress, genetics, environment and coexisting conditions [1]. Yet, while these advancements in understanding have indeed led to better treatment of heart failure, it remains a major cause of morbidity and mortality worldwide.

More recently, considerable evidence has shown that heart failure is associated with tissue ischemia and endothelial dysfunction, as assessed by impaired flow-mediated dilatation, as well as increases in specific plasma markers such as von Willebrand factor and soluble thrombomodulin [2]–[5]. A newer method to identify endothelial damage and dysfunction is the quantification of circulating endothelial cells (CECs) and endothelial progenitor cells (EPCs) in the peripheral circulation. CECs are mature endothelial cells that have detached from the intimal monolayer of blood vessels in response to endothelial injury [6], whereas EPCs are immature, bone-marrow derived cells with the capacity to transform into mature endothelial cells and promote postnatal angiogenesis and vasculogenesis [7]–[9]. EPCs can be characterized by the expression of surface markers, such as CD34, CD133 and VEGFR-2 (KDR or Flk-1) in various combinations [10]. It has, in fact, recently been shown by us that patients with heart failure have elevated circulating EPCs, which may be an independent predictor of mortality in CHF [11].

There are small membrane particles, known as endothelial microparticles, which are associated with endothelial cell damage and apoptosis. These endothelial microparticles have been shown to be elevated in conditions such as acute coronary syndrome (ACS) and myocardial infarction [12]–[14]. Recently, we identified, for the first time, a new population of apoptotic progenitor cells (APCs) which were elevated in patients with ACS [15]. The apoptotic progenitors can be divided into early, reversible apoptotic cells and late, irreversible apoptotic cells. In this study, we sought to quantify the number of apoptotic progenitor cells in patients with heart failure. In so doing, we learned that while CHF patients did not exhibit higher levels of total or early apoptotic progenitors than controls, the more severe CHF patients exhibited elevated numbers of late apoptotic progenitors compared to those with less severe CHF.

## Materials and Methods

### Study Subjects

We studied a total of 58 patients (median age 76.5) arbitrarily with various classes of heart failure according to the New York Heart Association (NYHA) classification. The control group comprised of 23 subjects with a similar profile of age (a median of 74 years, range 42–81), gender, a normal ejection fraction by echocardiography and no evidence of heart failure. The incidence of risk factors for atherosclerosis including diabetes, hypertension, smoking and treated hyperlipidemia did not differ between the study and control groups. With regard to medication use, study group patients had significantly increased use of warfarin and renal failure was more common as compared with controls. In addition, as seen in Table 1, we subdivided the 58 CHF patients into two subgroups: 33 patients with mild/moderate CHF (NYHA class I–II) and 25 patients with advanced CHF (NYHA class III–IV). There were no significant differences between the demographic characteristics of these two subgroups.

**Table 1:** Baseline characteristics CHF and control patients.

Group	Controls	NYHA I–II	NYHA III–IV	*P*
Characteristics	n = 23	n = 33	n = 25	
Demographic data				
Male/Female	15/8	25/8	18/7	
Median age (range)	74 (42–81)	73 (48–89)	74 (47–79)	ns
Current Smoker	2 (9%)	6 (18%)	4 (16%)	
Comorbidities				
Hypertension	12 (52%)	21 (63%)	14 (56%)	ns
Diabetes Mellitus	12 (52%)	20 (61%)	14 (56%)	ns
Hyperlipidemia	16 (65%)	26 (79%)	15 (60%)	ns
Drug Treatment				
Statin	16 (65%)	27 (82%)	13 (52%)	ns
Beta Blocker	6 (26%)	25 (76%)	19 (76%)	<.01
ACEI/ARB	16 (65%)	27 (82%)	19 (76%)	ns
Spironolactone	0	23 (70%)	17 (68%)	<.01
Furosemide	0	33 (100%)	25 (100%)	<.001 <.01
Coumadin	0	14 (42%)	10 (40%)	<.01
Measurements				
Ejection Fraction*				
>40%		13 (39%)	10 (40%)	
≤40%		20 (61%)	15 (60%)	
Creatinine				
>1.5		20 (61%)	16 (64%)	
≤1.5		13 (39%)	9 (36%)	
Ischemic vs. Non-IschemicΔ				
Ischemic			16 (64%)	
Non-ischemic			20 (36%)	

* LV ejection fraction was estimated by 2D echocardiography.

Δ Ischemic cardiomyopathy refers to patients with ischemic heart disease (prior MI, PTCA or CABG) and ejection fraction <40%.

Of the CHF patients, 43 were male and 15 were female. Of the controls, 15 were male and 8 were female. Table 1 summarizes demographic and clinical characteristics of the patient population. Institutional ethics committee approved the study and informed consent was obtained from all patients.

### Preparation of Blood Samples

Peripheral blood mononuclear cells (PBMNCs) were isolated from 20 ml of freshly drawn heparinized blood using Isopaque-Ficoll (Amersham Biosciences, Buckinghamshire, United Kingdom) gradient centrifugation.

### Flow Cytometry evaluation of early and late apoptotic progenitor CD34+ cells

After Ficoll gradient separation, PBMNCs were washed with phosphate-buffered saline (PBS), and 10^6^ cells were stained with (FITC)-anti-CD34 MAb for 30 minutes at 4°C in 100 microliters of FACS staining buffer (PBS and 2% fetal calf serum (FCS). Apoptosis in progenitor CD34+ progenitor cells was assessed using Southern Biotech ApoScreen Annexin V Apoptosis detection kit (Annexin V-PE, Propidium Iodide (PI) solution and Annexin V binding buffer). This assay involves staining peripheral blood mononuclear cells with Annexin V-PE (a phospholipid-binding protein binding to disrupted cell membranes) in combination with propidium iodide (PI, a vital dye binding to DNA penetrating into apoptotic cells). FACS analysis of CD34+ progenitor cells that are in early (annexin V+/PI−) or late (annexin V+/PI+) apoptotic phase was performed.

The percentage of apoptotic CD34+ progenitor cells (out of total circulating CD34+ progenitor cells) was assessed by staining peripheral blood mononuclear cells for 3 color FACS analysis employing (FITC)-anti-CD34 MAb (IQ products), Annexin V-PE and Propidium Iodide (SouthernBiotech). The cells were then washed again with PBS and resuspended in 100 microliters of Annexin V-PE binding buffer and incubated with 5 microliters of Annexin V-PE for 15 minutes at room temperature. Without washing, another 200 microliters of binding buffer and 5 microliters of PI solution were added, and 800,000 cells were acquired by flow cytometry (FACSCalibur, Becton Dickinson) and analyzed by CellQuest software (BD Bioscience). All analyses and readings were made by technicians who were blinded to the study questions.

### Determination of Erythropoietin, Thrombomodulin/CD141 and Antibodies to Oxidized LDL levels

ELISA kits were used to detect levels of Erythropoietin (Stem Cell Technologies) and Thrombomodulin/CD141 (Diaclone). Antibody to oxidative LDL levels were detected by ELISA method with anti-human antibody and solutions created in the laboratory [19].

### Statistical Analysis

All data was summarized and displayed as mean (SD) for the continuous variables and as number of patients plus the percentage in each group for categorical variables. The one-way Kolmogorov-Smirnov test was used to assess the distributions. Levels of late apoptotic cells could not be converted to normal distribution, thus we categorized the variables into tertiles.

For all categorical variables the Chi-Square statistics was used for assessing the statistical significance between the groups of CHF severity, while for all continuous variables, the independent samples one way ANOVA test was used. Regression analysis and analysis of variance for APCs by independent variables was performed in all cases.

All above analyses were considered significant at p<0.05 (two tailed). The SPSS 15 statistical package was used to perform all statistical evaluations (SSPS Inc., Chicago, IL, USA).

## Results

**Figure 1:**
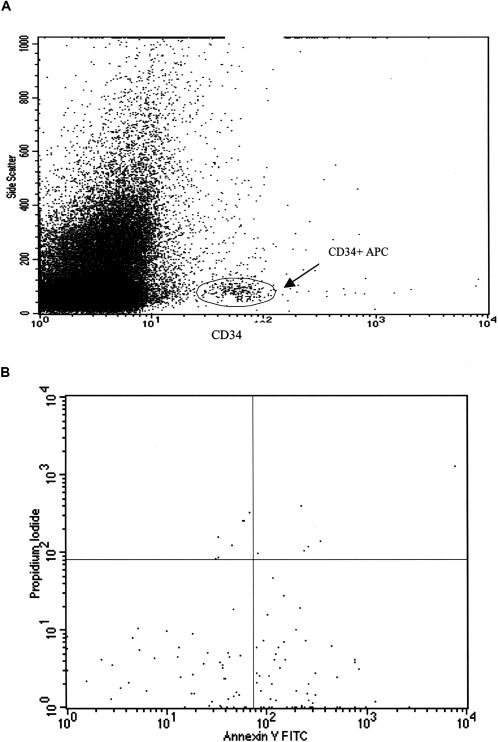
Representative flow cytometric dot plots: SSC/CD34 scatter (1a) and flow cytometric evaluation of progenitor CD34+ apoptotic cell percentage (1b). Dots in the lower right quadrant represent early apoptotic cells (Annexin-V positive and PI negative) while dots in the upper right quadrant represent late apoptotic cells (Annexin-V and PI positive).

We employed a novel assay for determining apoptotic CD34 cells. Progenitor CD34+ cells were initially gated from the side scatter/CD34 dot plot according to the Milan protocol [20]–[21]. As can be seen in Figure 1a, the progenitor CD34+ cells can be outlined and gated using this FACS dot plot. The percentage of apoptotic CD34+ progenitor cells was then determined by FACS analysis of Annexin V/PI staining. In apoptotic cells, the membrane phospholipids phosphatidylserine (PS) is translocated from the inner to the outer leaflet of the plasma membrane, thereby exposing PS to the extracellular environment. Annexin V is a 36 kDa Ca^2+^ dependent phospholipid-binding protein that has a high affinity for PS and binds to cells when PS is exposed to the external cellular environment, which occurs when membrane integrity is affected in early phase of apoptosis [16]–[17]. Propidium Iodide (PI) is a vital dye binding to DNA, a process implying disrupted cellular membrane and exposed DNA, compatible with late, irreversible cell necrosis. Cells that are viable are Annexin V-PE and PI negative; cells that are in early apoptosis are Annexin V-PE positive and PI negative (lower right quadrant–figure 1b); and cells that are in late apoptosis or necrosis are both Annexin V-PE and PI positive (upper right quadrant–figure 1b) [17]–[18].

**Figure 2:**
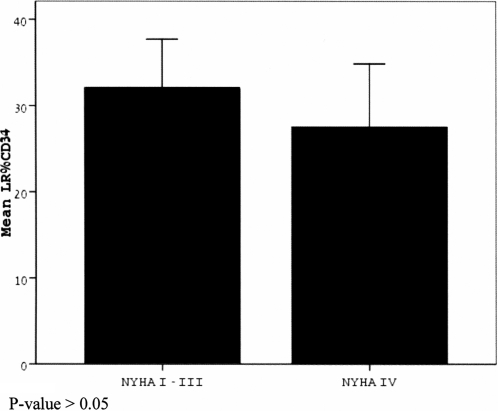
Early apoptotic progenitor CD34+ cell percentage (out of total circulating progenitor CD34+ cells) in mild/moderate and severe CHF groups. P-value is >0.05 after adjustment for variables.

**Figure 3:**
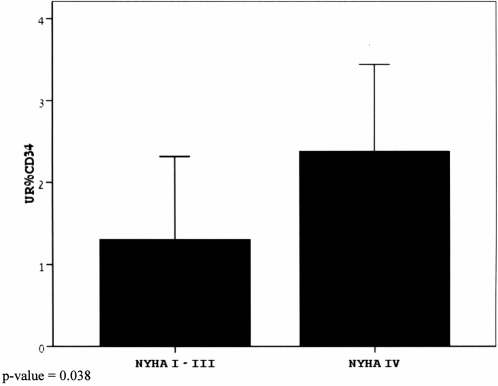
Late apoptotic progenitor CD34+ cell percentage (out of total circulating progenitor CD34+ cells) in mild/moderate (NYHA class 1–3) and severe (NYHA class 4) CHF groups. Chi-square P value of the distribution of tertiles between NYHA 1–3 & NYHA = 4 is 0.038.

As can be appreciated in Table 1, there were no significant statistical differences between the demographic characteristics of the two study groups (mild-moderate CHF and severe CHF) and the control group which comprised ACS patients not having CHF. With regard to the number of early apoptotic cells, no statistically significant difference was found between the age matched controls vs. CHF groups (once adjusted for age, gender and risk factors) or between the two CHF groups (mild/moderate vs. severe) (figure 2). Within the CHF groups, however, there was a positive correlation between the number of early apoptotic progenitor cells and levels of hemoglobin (r = 0.279, p = 0.016) and total CD34+ cells (r = 0.261, p = 0.023). With regard to the late apoptotic cells, there was an elevated number of cells in the severe CHF group compared to the mild/moderate CHF group (p =  0.013), as shown in figure 3. Interestingly, there was a positive association between late apoptotic cells and NYHA class (r = 0.223, p = 0.046) as well as a negative correlation between late apoptotic cells and ejection fraction (r = −0.252, p = 0.028). There was also a negative association between hyperlipidemia and total CD34+ progenitors in the CHF groups. Tables 2 and 3 demonstrate the parameters tested for correlations and associations. As shown in table 2, neither erythropoietin (r = 0.072; p = 0.3), thrombomodulin/CD141 (r = 0.09; p = 0.25), nor antibodies to oxidized LDL levels (r = −0.13; p = 0.19) correlated with early apoptotic progenitor cells.

**Table 2:** Correlations between early apoptotic/late apoptotic/total CD34+ EPCs and various parameters.

Parameter	Early Apoptotic Cells		Late Apoptotic Cells		Total CD34+ cells	
	r	p-value	r	p-value	r	p-value
Age	0.013	0.460	0.052	0.350	−0.123	0.180
Heart failure duration	−0.063	0.318	−0.018	0.447	−0.058	0.332
LVEF	−0.107	0.211	−0.252	0.028*	−0.152	0.127
Creatinine	0.033	0.403	−0.027	0.420	−0.049	0.357
Erythropoietin	0.072	0.298	0.157	0.123	0.031	0.409
Thrombomodulin	0.093	0.247	0.091	0.251	0.031	0.411
OxLDL antibodies	−0.117	0.194	−0.037	0.391	0.041	0.380
Hemoglobin	0.279	0.016*	−0.027	0.420	0.176	0.092
CD34% of total	0.261	0.023*	0.213	0.053		

* Statistically Significant.

**Table 3:** Associations between early apoptotic/late apoptotic/total CD34+ EPCs and various parameters.

Parameters	Early apoptotic cells		Late apoptotic cells		Total CD34+ cells	
	r	p-value	r	p-value	r	p-value
Gender	−0.027	0.420	−0.124	0.176	−0.214	0.053
Hyperlipidemia	0.130	0.165	−0.186	0.082	−0.236	0.038*
Smoking	0.094	0.241	−0.052	0.351	0.170	0.101
Hypertension	0.017	0.450	−0.074	0.291	−0.157	0.120
Diabetes Mellitus	0.108	0.210	−0.023	0.433	−0.113	0.200
Ischemic heart disease	0.040	0.384	−0.081	0.274	−0.106	0.215
TIA/CVA	0.065	0.313	0.174	0.096	−0.103	0.221
PTCA	0.109	0.209	−0.243	0.033*	0.124	0.178
CABG	0.071	0.300	0.028	0.417	−0.113	0.199
NYHA Class	−0.174	0.096	0.223	0.046*	0.098	0.232

* Statistically Significant.

## Discussion

Over the past decade, since the isolation of a circulating angioblast (later referred to as EPC) from adults with the capacity to differentiate into mature endothelial cells in response to ischemia [9], [22], much work has been done to further classify and characterize the role of endothelial progenitor cells. EPCs are released from the bone marrow in response to endothelial damage in order to facilitate in angiogenesis and vasculogenesis. Previous studies have shown elevated numbers of EPCs in ACS [23]–[25] as well as in heart failure [11], presumably due to the release of angiogenic factors and activation of multiple neurohormonal axes triggered by tissue ischemia [7]. It is also known that all major cardiovascular risk factors negatively influence these factors [26].

In CHF there is increased oxidative stress due to an imbalance between reactive oxygen species (including the superoxide anion, hydrogen peroxide, and the hydroxyl radical) and endogenous antioxidant defense mechanisms [32]. Oxidative stress may damage cellular proteins and cause myocyte apoptosis and necrosis. Markers of oxidative stress that are increased in CHF include plasma-oxidized low-density lipoproteins, malondialdehyde and myeloperoxidase (an index of leukocyte activation), urinary levels of biopyrrins (oxidative metabolites of bilirubin), and isoprostane levels in plasma and urine [32].

Endothelial cell (EC) apoptosis is an additional biomarker of endothelial damage and hemostasis that has more recently been explored [27]. The number of circulating endothelial microparticles positively correlates with the severity of coronary endothelial dysfunction, suggesting a close relationship between coronary endothelial-dependent vasodilation and EC apoptosis [12]. In addition, their functional properties such as their procoagulant activity, involvement in inflammation and direct effect on endothelial dysfunction play a large role [28]. We have recently described a novel assay to detect and quantify circulating apoptotic CD34+ progenitor cells showing that they were elevated in ACS patients compared with healthy controls [15].

Similar to ACS, CHF is associated with myocardial and peripheral tissue ischemia. Thus, CHF patients were found to exhibit endothelial dysfunction that is correlated with disease severity [29]. Previous studies have demonstrated elevated levels of EPCs in heart failure patients [11], yet levels of apoptotic EPCs in these patients have not been determined so far. In our study, we sought to quantify these apoptotic progenitor cells in patients with heart failure. In so doing, we divided these apoptotic cells into two groups: early, reversible apoptotic CD34+ cells and late, irreversible apoptotic progenitors. The late apoptotic progenitor cells represent cells whose plasma membrane is no longer intact. Our results show that, while there was no significant difference in numbers of early or late apoptotic cells between CHF patients and healthy controls, there was an elevated number of late apoptotic progenitors in the more severe CHF patients compared to the less severe heart failure patients. Furthermore, there was a negative correlation between late apoptotic progenitors and ejection fraction as well as a positive association between late apoptotic CD34 cells and NYHA class.

Several possible mechanisms could be postulated to explain these findings. Oxidative stress is known to cause endothelial dysfunction, and, as mentioned previously, CHF is associated with increased oxidative stress [32]. It has been demonstrated by Dernbach et al that EPCs are equipped with antioxidative enzyme systems, allowing for improved survival of cells undergoing severe oxidative stress [30]. Oxidized LDL has been shown to increase the rate of EPC senescence/apoptosis [31]. Antibodies to oxidized LDL are thought to mirror oxidative stress and have been shown to be increased in patients with CHF [19]. Although it would be plausible to explain the increase in late apoptotic cells in the more severe CHF (low EF and high NYHA class) patients as being due to increased oxidative stress, we did not find a statistically significant correlation between antibodies to oxidized LDL and apoptotic progenitor cells. Therefore, the relationship of additional indirect markers of oxidative stress which are known to be increased in CHF [32] should be further explored in future studies.

Erythropoietin and thrombomodulin have also been shown to play a role in endothelial function and heart failure. Both these markers, erythropoietin being a mobilizer of progenitor cells and thrombomodulin being associated with endothelial dysfunction, were not found to correlate with the number of apoptotic CD34 cells.

In view of the interplay between cytokines enhancing progenitor cell mobilization and those precipitating their apoptosis, our results support the hypothesis that increasing severity of heart failure shifts the balance towards enhanced progenitor cell apoptosis. The lower the ejection fraction, the poorer the forward flow, which may increase tissue ischemia and therefore endothelial damage.

It should be mentioned, however, that the relatively small number of circulating CD34+ cells poses a question as to the true functional importance of these cells and imposes a difficulty in determining their accurate number. In addition, our study is limited by the relatively small sample size.

In conclusion, we found that patients with advanced CHF have higher levels of late apoptotic progenitors than those with mild/moderate CHF and that levels of late apoptotic progenitors were positively associated with NYHA class and had a negative correlation with ejection fraction. These findings support the hypothesis that increasing severity of heart failure shifts the balance towards enhanced progenitor cell apoptosis. Therefore, apoptotic progenitor cells could be evaluated in future studies as a potential predictive biomarker in CHF.
